# Grand challenges in the field of stem cell research

**DOI:** 10.3389/fcell.2014.00002

**Published:** 2014-02-13

**Authors:** Atsushi Asakura

**Affiliations:** Stem Cell Institute, Paul and Sheila Wellstone Muscular Dystrophy Center, Department of Neurology, University of Minnesota Medical SchoolMinneapolis, MN, USA

**Keywords:** stem cell, regeneration, embryonic stem cell, induced pluripotent stem cell, satellite cell, hematopoietic stem cell, direct reprogramming, MyoD

## Discovery of adult stem cells

In 1961, Mauro first discovered that skeletal muscle fibers of frogs contain mononuclear cells underneath the basal lamina by electron microscope and termed these “satellite cells” (Mauro, 1961). By contrast, they failed to find any satellite-like cells in cardiac muscle after thorough search of electron micrographs. Since then, satellite cells have been confirmed to be a true stem cell for skeletal muscle (von Maltzahn et al., 2013). Adult skeletal muscle possesses extraordinary regeneration capability (Seale and Rudnicki, 2000). After exercise or muscle injury, large numbers of new muscle fibers are normally formed within a week because of expansion and differentiation of muscle satellite cells as a stem cell population for muscle regeneration. In the same year, Till and McCulloch first established the concept of stem cells using bone marrow transplantation into irradiated mice (Till and McCulloch, 1961; Armstrong et al., 2012). They found that donor-derived hematopoietic stem cells (HSCs) or progenitor cells can be colonized in the recipient spleen after transplantation. A single colony can give rise to multiple hematopoietic lineages as well as stem or progenitor cells, indicating the multipotent differentiation and self-renewal abilities for the HSCs or progenitor cells.

In the last two decades, experiments have established the existence of adult stem cells in most tissues that appear to have the ability to differentiate into specific cell types following injury as a means of regeneration *in vivo* (Barker et al., 2010). For example, neural stem cells (NSCs) from the central nervous system can generate multiple neural cell types to respond (Gage and Temple, 2013). Bone marrow contains two distinct stem cell types: marrow stromal cells and HSCs. HSCs possess their ability to produce all blood cell lineages (Ugarte and Forsberg, 2013). Multipotent mesenchymal stem cells derived from marrow stromal cells can differentiate into a wide variety of cell lineages, including skeletal muscle, neurons, and hematopoietic cells *in vitro* and following transplantation (Pittenger et al., 1999; Dimarino et al., 2013). Therefore, many or all tissues may contain a population of stem cells that can differentiate in an appropriate manner in response to the growth factors and signals provided by their host tissues. The current challenge is to understand the molecular mechanisms of how these adult stem cells maintain their stem cell qualities: specific differentiation capability, self-renewal activity, and response to the microenvironments of the stem cell niche.

## Discovery of embryonic stem cells

Embryonic stem cells (ESCs) were first isolated from the inner cell mass of a mouse blastocyst, an early-stage embryo, by Evans and Kaufman (1981). Since then, ESCs have been of particular interest to developmental biologists. This is because ESCs are pluripotent stem cells characterized by their ability to both self-renew and differentiate into multiple cell lineages, and the differentiation processes of ESCs can recapitulate embryonic development (Bibikova et al., 2008). In addition, their ability of pluripotency was utilized to create chimeric mice after blastocyst injection of ESCs, which allows us to manipulate gene modifications in living animals for things such as generating gene knockout mice and transgenic mice (Thomas and Capecchi, 1987). Following germ line transmission, the ESCs are able to contribute to whole animal bodies including germ cells (Capecchi, 1989). Finally, tetraploid embryo complementation experiments, which are the most stringent tests of full pluripotency, demonstrate that ESCs are a stem cell line sufficient for developmental potential to generate all tissues required for an organism (Nagy et al., 1990). Because of their pluripotent differentiation abilities, ESCs have been considered for clinical use in regenerative medicine after differentiation of ESCs into specific cell types such as liver cells, neurons, cardiomyocytes, and skeletal muscle (Ben-David et al., 2012). In 2007, Nobel Laureates (Capecchi and Evans) made a series of ground-breaking discoveries concerning ESCs and ESC-mediated DNA recombination in mice. The central challenge is to manipulate ES cells to induce tissue-specific cell types as well as complex functional organs *in vitro*, which is also crucial for cell-based therapy for regenerative medicine.

## Discovery of cell fate reprogramming by transcription factors

Our knowledge of the molecular mechanisms for stem cell differentiation was dramatically accelerated since the 1987 discovery of the MyoD-family of transcription factors, termed the myogenic regulatory factors (MRFs), by Weintraub group (Davis et al., 1987). The MRFs comprise a group of muscle-specific basic helix-loop-helix transcription factors, consisting of MyoD, Myf5, myogenin, and MRF4. The MRFs can initiate transcription of muscle-specific genes through direct binding to E-boxes, a consensus DNA motif, existing in the regulatory regions of these genes (Weintraub et al., 1991). Expression of a single MRF gene, *MyoD*, in various cell types, including fibroblasts, pigment, nerve, fat and liver cells, is able to initiate myogenic reprogramming (Weintraub et al., 1989). Therefore, the MyoD family is the first discovered master reprogramming factor. Thereafter, many scientists have successfully identified tissue-specific and pluripotent master reprogramming factors whose involvement includes neurogenesis and ES cells.

In 2006, Takahashi and Yamanaka first discovered that expression of the four transcription factors (Yamanaka factors), Oct4, Sox2, cMyc, and Klf4 generate induced pluripotent stem cells (iPSCs) from fibroblasts (Takahashi and Yamanaka, 2006). Currently, many types of somatic cells including B lymphocytes, natural killer T cells, stomach cells, liver cells, pancreatic β-cells, NSCs and skeletal myoblasts can be reprogrammed into iPSCs by expression of Yamanaka factors (Kim et al., 2010; Watanabe et al., 2011; Kumar et al., 2013). The iPSCs generated from adult tissues have been thought to mimic ESCs. Therefore, these Yamanaka factors are considered as pluripotent reprogramming factors. In addition, iPSCs hold tremendous therapeutic promise due to their ability to proliferate, differentiate, and self-renew. The quick generation of iPSCs from any somatic cells enables them to be a source of patient-derived cells for regenerative therapies after differentiation into particular cell types (Yoshida and Yamanaka, 2010). In 2012, Yamanaka and Gurdon were awarded the Nobel Prize for Physiology or Medicine for the discovery of generation of iPSCs and nuclear reprogramming; in both of which, mature somatic cells can be converted to pluripotent stem cells (Gurdon, 1962; Takahashi and Yamanaka, 2006; Yamanaka and Blau, 2010). However, there are several safety concerns such as the potential risk of tumorigenesis and the genetic and epigenetic aberrations after transplantation of iPSC-derived cells, partly diminishing the enthusiasm of using iPSC technology for mediated regenerative medicine in humans (Barrilleaux and Knoepfler, 2011). Direct reprogramming can overcome these difficulties since somatic cells can be directly reprogrammed into particular cell types without going through the iPSC or pluripotent stage. As mentioned above, MyoD is the first reprogramming factor for myogenesis. In the last few years, several papers reported the direct-reprogramming of fibroblasts into differentiated cell lineages including neurons, cardiomyocytes, hepatocytes, pancreatic β-cells, and blood cell progenitors by transduction of specific transcription factor cocktails (Chambers and Studer, 2011). Directed reprogramming technology will allow us to understand the molecular mechanisms of stem cell differentiation and de-differentiation as well as provide useful and safe cell sources for therapies of many regenerative medicines in humans (Morris and Daley, 2013). The generation of efficient, functional, tissue-specific cell types from iPSCs and other somatic cells remains a challenge.

The goal of *Stem Cell Research* is to publish all aspects of stem cell research ranging from molecular and cellular biology to developmental biology and tissue regeneration. In addition, the journal will consider papers in areas of the field in progenitor or stem cells related to cloning, pluripotency, reprogramming, proteomics, genetics, epigenetics, genomics, non-coding RNAs, and cancer stem cells. Our aim is to foster research that integrates all levels of stem cell biology, including developmental biology, tissue growth and regeneration, diseases, aging, and cancer. We welcome original papers on a broad range of subjects relating to stem cells, developmental biology, tissue regeneration, disease models, drug screening, and bioinformatics, related to stem cell biology and tissue regeneration.
